# Association of High-Density Lipoprotein Cholesterol with Macular Structure in Nonglaucomatous Individuals

**DOI:** 10.1016/j.xops.2026.101073

**Published:** 2026-01-14

**Authors:** Taiga Inooka, Ryo Tomita, Ayana Suzumura, Shota Fujikawa, Yuki Kimura, Taro Kominami, Tetsuhito Kojima, Shinji Ueno, Yasuki Ito, Koji M. Nishiguchi, Kenya Yuki

**Affiliations:** 1 Department of Ophthalmology, Nagoya University Graduate School of Medicine, Nagoya, Japan; 2 Aichi Health Promotion Foundation, Nagoya, Japan; 3 Department of Ophthalmology, Hirosaki University Graduate School of Medicine, Hirosaki, Japan; 4 Department of Ophthalmology, Fujita Health University School of Medicine, Toyoake, Japan

**Keywords:** HDL, Retinal ganglion cells, Tomography, Optical coherence, Adult

## Abstract

**Purpose:**

To investigate the association between serum high-density lipoprotein cholesterol (HDL-C) levels and ganglion cell complex (GCC) thickness in a nonglaucomatous Japanese population.

**Design:**

A retrospective cross-sectional observational study.

**Participants:**

We included 588 nonglaucomatous Japanese adults who underwent comprehensive ophthalmic and systemic health screening.

**Methods:**

Participants underwent OCT imaging, anthropometric measurements, including brachial–ankle pulse wave velocity, spirometry, and hematologic profiling. Multivariable linear regression models were used to assess the association between HDL-C levels and GCC thickness. Covariates were selected using a stepwise variable selection procedure, with the final model including age and axial length. A piecewise linear regression model further evaluated the association across different HDL-C ranges.

**Main Outcome Measures:**

Average GCC thickness.

**Results:**

Older age (*P* = 0.002), longer axial length (*P* < 0.001), and higher HDL-C levels (*P* < 0.001) were significantly associated with thinner GCC thickness. A nonlinear relationship was observed, with GCC thickness inversely associated with HDL-C levels outside the 60 to 67 mg/dL range (*P* = 0.005).

**Conclusions:**

High-density lipoprotein cholesterol levels are significantly associated with GCC thickness in nonglaucomatous individuals, which suggests a potential role of lipid metabolism in early neuroretinal thinning. High-density lipoprotein cholesterol may serve as a biomarker for neurodegenerative changes, even before glaucomatous alterations become clinically apparent.

**Financial Disclosure(s):**

Proprietary or commercial disclosure may be found in the Footnotes and Disclosures at the end of this article.

The neuroretina, which is an extension of the central nervous system, potentially reflects the status of systemic health functions.^1^, ^2^, ^3^ In the field of ophthalmology, advancements in OCT have highlighted the potential role of the ganglion cell complex (GCC), including the retinal nerve fiber layer (RNFL) and the ganglion cell-inner plexiform layer, as a unique marker of aging-related and common diseases.^4^, ^5^, ^6^, ^7^, ^8^, ^9^, ^10^, ^11^, ^12^, ^13^ Reports have emphasized the potential association between elevated high-density lipoprotein cholesterol (HDL-C) levels and various adverse health outcomes,^14^^,^^15^ particularly, in ophthalmology, where high HDL-C levels were associated with ocular conditions, such as elevated intraocular pressure (IOP), thinner RNFL, and the presence of glaucoma.^16^, ^17^, ^18^

Furthermore, lifestyle diseases, such as hyperglycemia,^19^ dyslipidemia,^20^ arteriosclerosis,^21^^,^^22^ and pulmonary airflow limitation^23^^,^^24^ could influence the GCC and ultimately affect the outcomes and progression of glaucoma. However, few studies have investigated the combined influence of these lifestyle disease-associated factors on the normal macular structure within the same population. Therefore, it is important to evaluate the effects of the HDL-C on individuals with normal macular structures as well as on lifestyle disease-associated factors. This evaluation is essential for contextualizing future research that explores the relationship between the macular structure and diseases.

Thus, this study was conducted with an aim to comprehensively assess the correlation between lifestyle disease-associated parameters and the GCC thickness, which was ascertained from ocular examination, hematologic tests, and anthropometric data, including volume plethysmography, all of which were obtained during health examinations of participants without glaucoma.

## Methods

### Study Design

This retrospective cross-sectional observational study, conducted at a single center, complied with the ethical principles outlined in the Declaration of Helsinki. The Institutional Review Board of Nagoya University Graduate School of Medicine approved the study protocol (Approval No. 2025-0237 37403). Informed consent was obtained using an opt-out approach, and written informed consent was obtained for the clinical screening itself as part of routine procedures. All participant-identifying information was removed before analysis.

### Participants

This retrospective study utilized data from health and eye screenings conducted between October 2022 and September 2023 at the Aichi Health Promotion Foundation (Aichi, Japan). The collected data included ophthalmic parameters, anthropometric assessments, hematological test results, and lifestyle questionnaires.

The inclusion criteria were (1) age >20 years, (2) refractive errors within 6 diopters and 3 diopters of spherical and cylindrical vision, (3) corrected visual acuity of 20/20 or better, and (4) participants without abnormal findings (including, but not limited to epiretinal membrane, macular hole, nerve fiber layer defect, and retinal pigment epithelium abnormalities) in the OCT undertaken in the screening and evaluated by ophthalmologists (T.I. and Y.K.). The exclusion criteria were (1) previous diagnosis of open-angle or angle-closure glaucoma; (2) IOP >21 mmHg; (3) history of any retinal or optic nerve disease that affects OCT measurements (including, but not limited to, age-related macular degeneration, uveitis, and optic neuritis); (4) history of ocular trauma or ocular surgery, including vitreous and corneal surgery; and (5) history of intracranial lesion, neurologic disorder, rheumatologic disease, or systemic vasculitis. To ensure data independence, 1 eye per participant was included. When both eyes qualified, the right eye's data were utilized. Participants identified as having abnormal results for any test in the health screening were directed to consult physicians of specialties for appropriate treatments.

### Ocular Examinations

All participants received complete ophthalmic assessments and concurrent hematological and anthropometric tests, which included volume plethysmography, on the same day. Corrected visual acuity, axial length, and IOP were measured using an automatic vision tester (Tomey CA-1000), a partial coherence interferometer (Tomey OA-2000), and a noncontact tonometer (Canon, TX-20P), respectively. The GCC thickness was measured using OCT (Canon, OCT-HS100). The device’s proprietary Glaucoma 3D analysis module automatically segments the macular inner retinal layers—specifically the RNFL, ganglion cell layer, and inner plexiform layer—and reports the overall average GCC thickness by averaging values across superior and inferior hemifields within a 10-mm-diameter circle on the macular scan.^25^ All data were evaluated by ophthalmologists (T.I. and Y.K.).

### Hematological, Volume Plethysmography, and Pulmonary Function Test

Blood was drawn after overnight fasting, and serum was used for standard biochemical testing in an accredited laboratory. In conformance with the criteria set by the NCEP ATP III,^26^ lipid-related variables included total cholesterol, triglyceride (TG), low-density lipoprotein cholesterol (LDL-C), and HDL-C. Based on the Japanese clinical practice guidelines for diabetes,^27^ the fasting blood sugar and glycated hemoglobin levels were used in the analysis as factors that are associated with glycemia.

Brachial–ankle pulse wave velocity (baPWV) was measured via an automated volume plethysmography (Omron HealthCare, BP-203RPEIII), following a 10-minute resting period in the supine position. Trained personnel applied cuffs to the arms and ankles, and the device computed the transit time and travel path that was extrapolated from the participant's height to derive baPWV values. Higher baPWV values measured on either side of each participant were used for the analysis. Moreover, the device simultaneously measured the blood pressure (BP), including the systolic BP and diastolic BP.

For pulmonary function, spirometry (Vmax 22, Sensor-Medics) was performed by trained technicians following current recommendations.^28^ Spirometry test produced forced expiratory volume in 1 second (FEV_1_) and forced vital capacity (FVC). According to the GOLD criteria,^29^ the percentage of predicted FEV_1_ (%FEV_1_) was used as a representative index of pulmonary function in the analysis.

### Statistical Analyses

For statistical analyses, parametric variables were presented as mean ± standard deviation, nonparametric variables as median (interquartile range: 25th percentile to 75th percentile), and categorical variables as the frequency (percentage). The Shapiro–Wilk test was conducted to determine the distribution of data, and a logarithmic transformation was performed for non-normally distributed data. Decimal visual acuity values were converted to the logarithm of the minimum angle of resolution units. Body mass index (BMI) was calculated from measured height and weight as weight (kg) divided by height squared (m^2^). Smoking history was binarized as ever-smoker (current or former) versus never, and alcohol consumption was coded as an ordinal variable with three categories (none, occasional, and daily).

Multivariable linear regression models were constructed using a stepwise selection method, guided by the Akaike information criterion. In case GCC thickness was designated as the response variable, explanatory variables comprised age, sex, BMI, smoking history, alcohol consumption, IOP, axial length, baPWV, systolic BP, diastolic BP, several hematologic test results (total cholesterol, TG, LDL-C, HDL-C, fasting blood sugar, and glycated hemoglobin), and %FEV_1_. In case HDL-C level was designated as the response variable, explanatory variables comprised age, sex, BMI, smoking history, alcohol consumption, IOP, axial length, baPWV, systolic BP, diastolic BP, the hematologic test results, and %FEV_1_. To enable the direct comparison of effect sizes across variables, standardized partial regression coefficients (β) were calculated by normalizing all predictors to have a mean of zero and a standard deviation of 1. The final model's results, obtained via backward stepwise selection, are reported using unstandardized partial regression coefficients (B) and their standard errors. Multicollinearity severity was estimated by calculating variance inflation factors. A *P* value of <0.05 was considered statistically significant.

To visualize trends between HDL-C levels and GCC thickness, locally weighted scatterplot smoothing curves were fit. Locally weighted scatterplot smoothing visualization suggested relatively linear slopes with two knots in adjacent HDL-C level epochs. Therefore, piecewise linear regression models that incorporated two knots were employed to better characterize the segmented association between HDL-C and GCC thickness. In the piecewise linear regression analyses, univariable regression analyses were used, and multivariable linear regression analysis was used after adjusting for parameters statistically significantly correlated with GCC thickness in the multivariable linear regression model.

To ensure robustness of the associations, we conducted two sensitivity analyses: (1) replacing %FEV_1_ with a dichotomous airway-obstruction indicator defined as FEV_1_/FVC <0.70 versus ≥0.70, and (2) excluding participants treated for dyslipidemia to account for the effect of statin users (124 participants excluded). All analyses were performed using scikit-learn 0.24.0, based on Python 3.6.7 and R 4.2.2 (R Foundation for Statistical Computing).

## Results

### Demographic Information

A total of 588 eyes were used for statistical analyses. The main demographic characteristics of the participants are presented in Table 1.

**Table 1:** Demographic Information of the Participants

Number of Eyes	588
Sex (Male/Female)	430/158
Age (years)	54.9 ± 10.4 [25.0, 84.0]
BMI (kg/m^2^)	23.2 (21.4, 26.0) [13.8, 37.0]
History of smoking (never/current or former), n (%)	471 (80.1%)/117 (19.9%)
History of alcohol intake (none/sometimes/daily), n (%)	182 (31.0%)/204 (34.7%)/202 (34.4%)
CVA (logMAR units)	–0.1 (–0.2, 0.0) [–0.2, 0.0]
IOP (mmHg)	13 (12, 15) [8, 21]
Axial length (mm)	24.3 (23.6, 25.3) [20.8, 28.6]
Average GCC thickness (μm)	93.0 (89.5, 97.0) [75.5, 112.5]
Total cholesterol (mg/dL)	198 (176, 222) [106, 320]
TG (mg/dL)	91 (66, 137) [28, 734]
LDL-C (mg/dL)	124 (106, 144) [46, 232]
HDL-C (mg/dL)	60 (51, 71) [30, 119]
BS (mg/dL)	99 (93, 107) [65, 260]
HbA1c (%)	5.6 (5.4, 5.9) [4.7, 11.2]
sBP (mmHg)	120.0 (111, 129) [82, 205]
dBP (mmHg)	75 (68, 84) [43, 127]
baPWV (cm/s)	1415.5 (1253.8, 1603.2) [913.0, 3563.0]
%FEV_1_	94.1 (85.1, 102.1) [37.7, 144.7]
FEV_1_/FVC (%)	79.2 (75.4, 82.9) [44.6, 97.6]

baPWV = brachial–ankle pulse wave velocity; BMI = body mass index; BS = blood sugar; CVA = corrected visual acuity; dBP = diastolic blood pressure; FEV_1_ = forced expiratory volume in 1 second; FVC = forced vital capacity; GCC = ganglion cell complex; HbA1c = glycated hemoglobin; HDL-C = high-density lipoprotein cholesterol; IOP = intraocular pressure; LDL-C = low-density lipoprotein cholesterol; logMAR = logarithm of the minimum angle of resolution; sBP = systolic blood pressure; TG = triglyceride.

Data are mean ± standard deviation [range] for parametric variables, median (interquartile range: 25th percentile–75th percentile) [range] for nonparametric variables, or frequency (percentage) for categorical variables.

### Correlations between the GCC Thickness and Health Checkup Factors

The Shapiro–Wilk test indicated that GCC thickness was not normally distributed (*P* < 0.001); therefore, logarithmic transformation was performed on the GCC thickness (*P* = 0.073, after logarithmic transformation). Multivariable linear regression with a stepwise selection method showed that older age (*P* = 0.002), longer axial length (*P* < 0.001), and higher HDL-C levels (*P* < 0.001) significantly correlated with thinner logarithms of the GCC thickness (Table 2).

**Table 2:** Multivariable Linear Regression Analysis between Logarithm GCC Thickness and Explanatory Variables

	Standardized Partial Regression Coefficient (β)	Partial Regression Coefficient (B)	Standard Error	*P* Value	VIF
Age (years)	–0.129	–7.6 × 10^–4^	2.4 × 10^–4^	0.002∗	1.07
BMI (kg/m^2^)	–0.082	–1.4 × 10^–3^	7.7 × 10^–4^	0.073	1.30
Axial length (mm)	–0.183	–9.3 × 10^–3^	2.1 × 10^–3^	<0.001∗	1.05
HDL-C (mg/dL)	–0.184	–7.5 × 10^–4^	1.9 × 10^–4^	<0.001∗	1.37
%FEV_1_	0.076	3.4 × 10^–4^	1.8 × 10^–4^	0.064	1.06

BMI = body mass index; FEV_1_ = forced expiratory volume in 1 second; GCC = ganglion cell complex; HDL-C = high-density lipoprotein cholesterol; VIF = variance inflation factor.

∗ *P* < 0.05.

**Figure 1:**
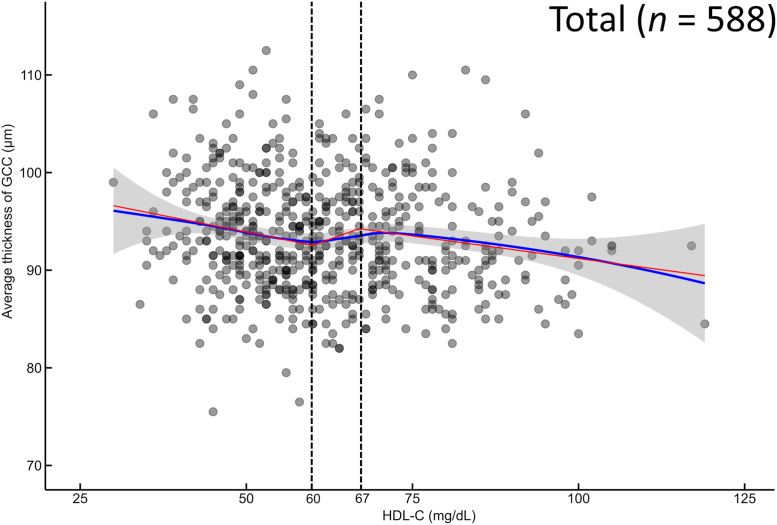
The LOESS curves of the GCC thickness as a function of the HDL-C levels. The blue line represents the LOESS curve, and the red line represents the piecewise linear regression models with two knots. The dashed lines delineate segments for regression analysis, which indicate potential shifts in trends. GCC = ganglion cell complex; HDL-C = high-density lipoprotein cholesterol; LOESS = locally estimated scatterplot smoothing.

Figure 1 shows the locally weighted scatterplot smoothing curve, which depicts the trend in average GCC thickness across a range of HDL-C levels. The GCC thickness decreased until approximately 60 mg/dL HDL-C and stabilized thereafter without any significant decreasing trend, followed by a small peak at approximately 67 mg/dL, and by a downward trend. Therefore, piecewise linear regression was performed with knots of 60 and 67 mg/dL. No significant correlation was found with the average GCC thickness in the HDL-C range of 60 to 67 mg/dL (*P* = 0.217); however, the HDL-C level was significantly negatively associated with the GCC thickness when the HDL-C level was <60 or >67 mg/dL (both *P* = 0.005) after adjusting for age and axial length (Table 3).

**Table 3:** Results of the Piecewise Linear Regression Analysis of the Association between the GCC Thickness and HDL-C Level

Segment of HDL-C, mg/dL	Univariable Analyses		Multivariable Analysis	
	Partial Regression Coefficient (B) (95% CI)	*P* Value	Partial Regression Coefficient (B) (95% CI)	*P* Value
Segment 1, <60 mg/dL	–0.13 (–0.26, –0.06)	0.002∗	–0.14 (–0.24, –0.05)	0.005∗
Segment 2, 60–67 mg/dL	+0.24 (–0.15, +0.69)	0.210	+0.27 (–0.16, +0.70)	0.217
Segment 3, >67 mg/dL	–0.09 (–0.18, –0.02)	0.012∗	–0.11 (–0.18, –0.03)	0.005∗

CI = confidence interval; GCC = ganglion cell complex; HDL-C = high-density lipoprotein cholesterol.

In the multivariable analyses, they were adjusted for age and axial length.

∗ *P*<0.05.

### Correlations between the HDL-C and Health Checkup Factors

The Shapiro–Wilk test indicated that the HDL-C level was not normally distributed (*P* < 0.001); therefore, logarithmic transformation was performed on the HDL-C level (*P* = 0.099, after logarithmic transformation). Multivariable linear regression with a stepwise selection method showed that female sex (*P* < 0.001), more frequent alcohol intake (sometimes, *P* = 0.005 and daily, *P* < 0.001), higher baPWV (*P* = 0.019), higher total cholesterol, lower TG, and lower LDL-C (all *P* < 0.001) were significantly correlated with higher logarithmic HDL-C levels (Table 4).

**Table 4:** Multivariable Linear Regression Analysis of the Association between the Logarithmic HDL-C and Explanatory Variables

	Standardized Partial Regression Coefficient (β)	Partial Regression Coefficient (B)	Standard Error	*P* Value	VIF
Sex (Male 0/Female 1)	7.5 × 10^–2^	4.0 × 10^–2^	8.7 × 10^–3^	<0.001∗	1.20
Age (years)	–3.4 × 10^–2^	–7.8 × 10^–4^	4.0 × 10^–4^	0.052	1.30
Frequency of alcohol intake (None 0/Sometimes 1)	NA	2.3 × 10^–2^	8.1 × 10^–3^	0.005∗	1.04
Frequency of alcohol intake (None 0/Daily 1)	NA	6.3 × 10^–2^	8.5 × 10^–3^	<0.001∗	1.04
IOP (mmHg)	2.3 × 10^–2^	2.2 × 10^–3^	1.3 × 10^–3^	0.099	1.03
Total cholesterol (mg/dL)	1.40	9.8 × 10^–3^	1.9 × 10^–4^	<0.001∗	1.99
TG (mg/dL)	–0.64	–2.0 × 10^–3^	5.1 × 10^–5^	<0.001∗	1.22
LDL-C (mg/dL)	–1.19	–9.8 × 10^–3^	2.1 × 10^–4^	<0.001∗	1.91
FBS (mg/dL)	2.4 × 10^–2^	3.0 × 10^–4^	2.1 × 10^–4^	0.149	1.20
baPWV (cm/s)	4.1 × 10^–2^	3.2 × 10^–5^	1.4 × 10^–5^	0.019∗	1.29

baPWV = brachial–ankle pulse wave velocity; FBS = fasting blood sugar; HDL-C = high-density lipoprotein cholesterol; IOP = intraocular pressure; LDL-C = low-density lipoprotein cholesterol; NA = not available; TG = triglyceride; VIF = variance inflation factor.

For categorical predictors, standardized partial regression coefficients are not shown because they depend on the chosen coding and category prevalence.

∗ *P* < 0.05.

### Sensitivity Analyses

In the sensitivity analysis that replaced %FEV_1_ with a dichotomous airway-obstruction indicator defined as FEV_1_/FVC <0.70 versus ≥0.70, 45 participants met the obstruction criterion (FEV_1_/FVC <0.70). The associations between HDL-C and GCC thickness were consistent with the main analysis; older age (*P* = 0.002), longer axial length (*P* < 0.001), higher HDL-C levels (*P* < 0.001), and presence of airway obstruction (*P* = 0.038) were significantly correlated with thinner logarithms of the GCC thickness in the multivariable linear regression analysis (Table S1, available at www.ophthalmologyscience.org). Piecewise regression confirmed the pattern—no association within 60 to 67 mg/dL (*P* = 0.259) and significant negative slopes below 60 and above 67 mg/dL (*P* = 0.001 and 0.002, respectively)—after adjusting for age, axial length, and presence of airway obstruction (Table S2, available at www.ophthalmologyscience.org).

In the other sensitivity analysis, which was conducted after excluding participants treated for dyslipidemia (124 participants were excluded), the adjustment between the average GCC thickness and HDL-C was also consistent; older age (*P* < 0.001), higher IOP (*P* = 0.023), longer axial length (*P* < 0.001), and higher HDL-C levels (*P* < 0.001) were significantly correlated with thinner logarithms of the GCC thickness in the multivariable linear regression analysis (Table S3, available at www.ophthalmologyscience.org). After adjusting for age, IOP, and axial length, the HDL-C level was significantly negatively associated with GCC thickness when the HDL-C level was <60 or >67 mg/dL (*P* < 0.001 and *P* = 0.005, respectively); however, no significant correlation was found with the GCC thickness within the HDL-C range of 60 to 67 mg/dL (*P* = 0.413; Fig S1 and Table S4, available at www.ophthalmologyscience.org).

## Discussion

This study revealed a potential association between high HDL-C levels (<60 or >67 mg/dL) and reduced GCC thickness. To identify this association, we considered ophthalmic findings, including axial length and IOP, and hematologic tests to detect abnormalities, such as hyperglycemia and dyslipidemia. Furthermore, we considered physical findings, including spirometry profiling, volume plethysmography, and BP. Based on these considerations, we found that the association between high HDL-C levels and reduced GCC thickness remained consistent. The study population was of mainland Japanese descent, with nonglaucomatous participants in the age range of 25 to 84 years. Our findings support and further extend the evidence of the previously reported association between high HDL-C levels and thinner macular structure,^18^ whereas a previous study focused on a relatively narrow age range (40–70 years) and predominantly European descent.

In the multivariable linear regression analysis, older age and longer axial length were significantly correlated with thinner GCC. Many previous studies have suggested an association between a thinner GCC and increasing age^4^^,^^30^, ^31^, ^32^ or longer axial length.^33^, ^34^, ^35^, ^36^, ^37^, ^38^, ^39^ In our study, a higher HDL-C level was associated with a thinner GCC, even when taking factors such as age, axial length, and other lifestyle disease-associated parameters into consideration. Though a higher HDL-C level correlated with the female sex, higher baPWV, and lipid-related parameters, including total cholesterol, TG, and LDL-C, in this study, none of the abovementioned parameters statistically correlated with GCC thickness. A previous report^22^ suggested that a higher pulse wave velocity may play a role in the damage to the macular ganglion cell-inner plexiform layer in patients with open-angle glaucoma; however, such an association was limited to patients whose baPWV exceeded 1800 cm/s on either side. By comparison, respiratory function showed a modest yet distinctive signal: while %FEV_1_ did not reach statistical significance with GCC thickness (*P* = 0.064), a dichotomous airflow-obstruction indicator defined as FEV_1_/FVC <0.70 was significantly associated with thinner GCC (*P* = 0.038), suggesting that clinically apparent airflow limitation may contribute to inner retinal thinning. This interpretation is consistent with previous reports in chronic obstructive pulmonary disease demonstrating inner retinal thinning on OCT.^23^^,^^24^^,^^40^ In contrast, a consistently significant correlation between HDL-C levels and the GCC thickness was detected on multivariable linear regression analyses. Thus, the HDL-C level may constitute a unique potential marker that correlates with the GCC thickness in a population without glaucomatous changes.

Interestingly, while higher HDL-C was associated with lower TGs and LDL-C and with more frequent alcohol intake, findings consistent with a metabolically favorable profile, HDL-C levels were also positively associated with higher baPWV, a marker of greater systemic arterial stiffness (Table 4). This paradoxical association supports the hypothesis that elevated HDL-C in this cohort may not necessarily reflect superior vascular health but could represent dysfunctional HDL with impaired vascular-protective capacity. Traditionally, owing to its close connection with a reduced risk of cardiovascular disease, HDL-C has been labeled as the “good cholesterol,”^41^ and some studies showed lower levels in primary open-angle glaucoma^42^, ^43^, ^44^, ^45^ or young patients with normal-tension glaucoma^46^ as compared with controls; however, other studies found no difference in the HDL-C levels.^20^^,^^47^^,^^48^ Even in recent studies focused on nonglaucomatous participants, findings have not been uniform: analysis based on macular RNFL showed that higher HDL-C is associated with thinner inner retinal structure,^18^ whereas an ETDRS grid–based report in a Japanese screening population found higher HDL-C in eyes with thicker inner-nasal and inner-inferior full-thickness sectors.^49^ We suggest that two complementary considerations help reconcile these observations without invoking contradictions in biology. First, the conflicting evidence for the association between the HDL-C level and glaucoma or retinal structure may be attributable to the relatively negative but nonlinear correlation between the HDL-C and retinal structure in healthy subjects. This inference is derived from a report of a negative correlation between superior retinal RNFL thickness and HDL-C levels in nonproliferative diabetic retinopathy.^50^ Furthermore, a large cohort study that included more than 400 000 UK Biobank participants demonstrated that elevated HDL-C levels were associated with an increased risk of glaucoma.^51^ Thus, a high HDL-C level may result in fundus findings of potential thinning of retinal nerve fiber structures in populations in the prefoveal stage, such as the nonglaucomatous population in our study and the nonproliferative diabetic retinopathy population in a previous study.^50^ Second, differences in the measured outcome—particularly the retinal layers they emphasize—may determine the direction of the observed association. Within this layer-specific balance, vascular effects that favor thickening and neuroaxonal effects that favor thinning can influence retinal structure in opposite directions. Accordingly, the direction of the association observed across studies depends on which of these opposing influences predominates in a given cohort and is further shaped by endpoint selection and its operational definition (e.g., full-thickness measurements versus GCC or RNFL). Full-thickness metrics aggregate signals from mid-to-outer retinal layers (inner nuclear, outer plexiform, Henle fiber, and outer nuclear layers), which are particularly susceptible to vasculometabolic influences such as subtle fluid shifts or extracellular matrix remodeling^52^ and may therefore manifest as sectoral thickening. By contrast, inner-retinal metrics, such as GCC and RNFL thickness, better index neuroaxonal integrity^53^^,^^54^ and thus preferentially detect thinning when neurodegenerative processes predominate. The direct connection may reflect the association between HDL-C levels and retinal markers of neurodegenerative diseases.^55^, ^56^, ^57^ Though the precise mechanisms and cellular pathways of lipid metabolism in neurodegenerative and retinal diseases are not yet fully elucidated, several hypotheses have been suggested. For instance, if an increase in HDL-C is attributable to impaired HDL-C processing or dysfunctional HDL-C, this may indicate a fundamental problem in cholesterol homeostasis that promotes oxidative damage and inflammation.^58^ Furthermore, variations in HDL apolipoprotein composition possibly alter the metabolic state and inflammatory response of microglia and thus potentially influence the degree of inflammatory damage to retinal ganglion cells.^59^^,^^60^ Accordingly, future studies should evaluate not only HDL-C concentration but also the quality and oxidative remodeling of HDL. Specifically, assessing cholesterol efflux capacity, myeloperoxidase and paraoxonase-1 activities, and oxidized lipid markers (e.g., oxidized LDL and 7-ketocholesterol) would help elucidate whether myeloperoxidase-mediated oxidation of apoA-I/HDL impairs cholesterol efflux, endothelial function, and paraoxonase-1 activity, as suggested in previous reports.^61^, ^62^, ^63^ A relevant clinical phenotype is the SCARB1 loss-of-function variant (P376L), in which high HDL-C levels paradoxically coexist with increased vascular risk,^64^ serving as a human model of high but dysfunctional HDL. Integrating these systemic indicators with OCT-based retinal phenotyping may help determine whether HDL-C–associated GCC thinning reflects HDL dysfunction rather than serum concentration alone.

In this study, the HDL-C level was significantly negatively associated with the GCC thickness when the HDL-C level was <60 mg/dL (1.6 mmol/L) or >67 mg/dL (1.7 mmol/L). The range in which no negative correlation between HDL-C levels and macular structure was observed differed from those in a previous report (1.6–1.7 and 1.3–1.7 mmol/L, in this and a previous study, respectively^18^). This discrepancy may be attributable to differences in the distribution of HDL-C values by ethnicity, as Japanese people have higher HDL-C values compared to individuals of British origin (median HDL-C 60 mg/dL [1.6 mmol/L] and 1.5 mmol/L in this and a previous study, respectively^18^^,^^53^). There are several possible reasons for the lack of significant correlation with the average GCC thickness and the HDL-C of 60 to 67 mg/dL (1.6–1.7 mmol/L, *P* = 0.413) in our study, or 1.3 to 1.7 mmol/L, in a previous study^18^; in this HDL-C range, the correlation between the HDL-C and the GCC thickness differed by sex; moreover, some degree of neuroprotective effect at moderate HDL-C levels may have obscured a unique trend.

This study had some limitations. First, no causal relationship between the GCC thickness and HDL-C level could be ascertained because of this study’s cross-sectional design, especially as no longitudinal analyses were performed. Second, the findings may have limited generalizability because the study sample consisted exclusively of Japanese individuals from a single city. Third, although results were consistent after excluding participants treated for dyslipidemia, there were limitations in the ascertainment of exposures and covariates that could not be fully addressed: (1) medical history relied on a self-reported questionnaire, which impeded comprehensive identification of prior therapeutic interventions, including medication classes and minor surgeries; detailed medication data were not available, precluding precise adjustment for HDL-modifying drugs or independent verification of lipid-lowering therapy; (2) BMI was included as an anthropometric indicator but does not necessarily reflect dietary intake, nutritional status, or physical activity;^65^ and (3) central corneal thickness and visual field testing were not available as part of this health check-up program, limiting the ability to fully exclude preperimetric glaucoma. In addition, IOP was measured using a noncontact tonometer (TX-20P; Canon). The TX-20P shows a strong correlation with Goldmann applanation tonometry,^66^ and noncontact tonometry has been widely used in population-based studies in which IOP was a primary endpoint.^67^^,^^68^ Nevertheless, systematic differences relative to Goldmann applanation tonometry cannot be excluded. Taken together, some influence from unmeasured or incompletely measured factors cannot be excluded. Finally, we did not correct for ocular magnification related to axial length on OCT. In this study, a longer axial length was associated with a thinner GCC; however, this may simply reflect the difference in the range of the image captured owing to the difference in the magnification rate. Nonetheless, the effect of HDL-C on GCC was evident, even after accounting for the effect of axial length.

## Conclusions

This study demonstrated that high HDL-C levels could be a potential biomarker of a thinner GCC in nonglaucomatous individuals, even after considering various parameters, such as age, pulse wave tests, and axial length. Thus, HDL-C may play a unique role in neurodegenerative conditions. These findings will contribute to the implementation of preventive interventions and improvement of patient outcomes.
