# Supplementary material for: Association of High-Density Lipoprotein Cholesterol with Macular Structure in Nonglaucomatous Individuals
Source: Ophthalmol Sci. 2026 Jan 14;6(3):101073. doi: 10.1016/j.xops.2026.101073 (PMC12907079; doi:10.1016/j.xops.2026.101073)
Supplement: Figure S1 [file mmc1.pdf]

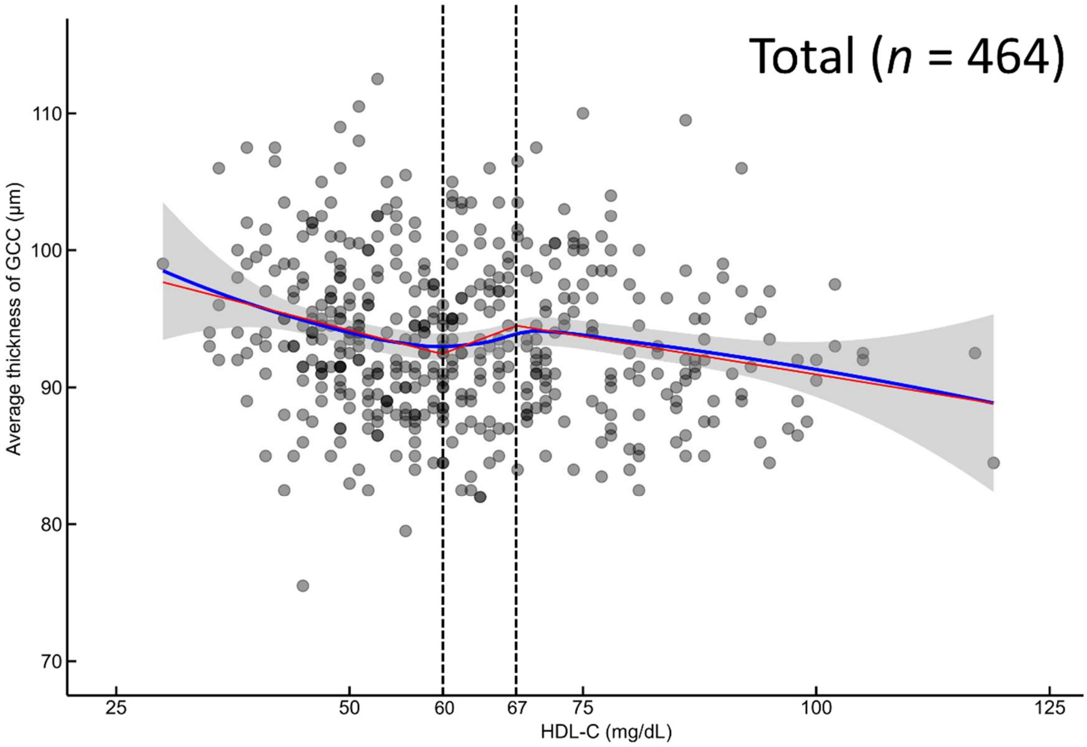

Supplementary Figure S1. **The LOESS curves of the GCC thickness as a function of the HDL-C levels, after excluding participants who received treatment for dyslipidemia.** The blue line represents the LOESS curve and the red line represents piecewise linear regression models with two knots. The dashed lines delineate segments for regression analysis, which indicates potential shifts in trends. LOESS, locally estimated scatterplot smoothing; HDL-C, high density lipoprotein cholesterol; GCC, ganglion cell complex.
