# Supplementary material for: Association of High-Density Lipoprotein Cholesterol with Macular Structure in Nonglaucomatous Individuals
Source: Ophthalmol Sci. 2026 Jan 14;6(3):101073. doi: 10.1016/j.xops.2026.101073 (PMC12907079; doi:10.1016/j.xops.2026.101073)
Supplement: Table S1 [file mmc2.pdf]

Supplementary Table S1. Multivariable linear regression analysis between logarithm GCC thickness and explanatory variables, after replacing %FEV_1 with a dichotomous airway-obstruction indicator defined as FEV_1/FVC <0.70 versus ≥0.70

	Standardized partial regression coefficient (β)	Partial regression coefficient (B)	Standard error	*P*-value	VIF
Age (years)	−0.131	-7.1×10^{-4}	2.5×10^{-4}	0.002*	1.11
BMI (kg/m²)	−0.078	-1.3×10^{-3}	4.6×10^{-2}	0.091	1.34
IOP (mmHg)	−0.059	-1.5×10^{-3}	1.0×10^{-3}	0.149	1.04
Axial length (mm)	−0.192	-9.7×10^{-3}	2.1×10^{-3}	<0.001*	1.06
HDL-C (mg/dL)	−0.172	-7.0×10^{-4}	1.9×10^{-4}	<0.001*	1.34
Presence of airway obstruction	−0.084	-1.9×10^{-2}	9.4×10^{-3}	0.038*	1.04

BMI = body mass index; VIF = variance inflation factor; IOP = intraocular pressure; HDL-C = high-density lipoprotein cholesterol.

Presence of airway obstruction was coded as 0 for FEV_1/FVC <0.70 and 1 for FEV_1/FVC ≥0.70.

*p <0.05
