# Supplementary material for: Association of High-Density Lipoprotein Cholesterol with Macular Structure in Nonglaucomatous Individuals
Source: Ophthalmol Sci. 2026 Jan 14;6(3):101073. doi: 10.1016/j.xops.2026.101073 (PMC12907079; doi:10.1016/j.xops.2026.101073)
Supplement: Table S2 [file mmc3.pdf]

Supplementary Table S2. Results of the piecewise linear regression analysis of the association between the GCC thickness and HDL-C level, after replacing %FEV_1 with a dichotomous airway-obstruction indicator defined as FEV_1/FVC <0.70 versus ≥0.70

Parameters	Univariable analyses		Multivariable analysis	
Segment of HDL-C, mg/dL	Partial regression coefficient (B) (95% CI)	*P*-value	Partial regression coefficient (B) (95% CI)	*P*-value
Segment 1, <60 mg/dL	−0.13 (−0.26, −0.06)	0.002*	−0.17 (−0.27, −0.07)	0.001*
Segment 2, 60–67 mg/dL	+0.24 (−0.15, +0.69)	0.210	+0.25 (−0.19, +0.69)	0.259
Segment 3, >67 mg/dL	−0.09 (−0.18, −0.02)	0.012*	−0.12 (−0.20, −0.05)	0.002*

HDL-C = high-density lipoprotein cholesterol; GCC = ganglion cell complex; CI = confidence interval.

In the multivariable analyses, they were adjusted for age, axial length, and presence of airway obstruction.

*p <0.05
