# Supplementary material for: Association of High-Density Lipoprotein Cholesterol with Macular Structure in Nonglaucomatous Individuals
Source: Ophthalmol Sci. 2026 Jan 14;6(3):101073. doi: 10.1016/j.xops.2026.101073 (PMC12907079; doi:10.1016/j.xops.2026.101073)
Supplement: Table S3 [file mmc4.pdf]

Supplementary Table S3. Multivariable linear regression analysis between logarithm GCC thickness and explanatory variables, after excluding participants who received treatment for dyslipidemia

	Standardized partial regression coefficient (β)	Partial regression coefficient (B)	Standard error	*P*-value	VIF
Age (years)	-1.4×10^{-2}	−0.13	3.2×10^{-2}	<0.001*	1.61
IOP (mmHg)	-6.3×10^{-3}	−0.24	0.11	0.023*	1.02
Axial length (mm)	-1.6×10^{-2}	−1.21	0.22	<0.001*	1.06
baPWV (cm/s)	5.5×10^{-3}	1.8×10^{-3}	1.1×10^{-3}	0.110	1.54
HDL-C (mg/dL)	-9.3×10^{-3}	-5.9×10^{-2}	1.7×10^{-2}	<0.001*	1.04

VIF = variance inflation factor; IOP = intraocular pressure; baPWV = brachial–ankle pulse wave velocity; HDL-C = high-density lipoprotein cholesterol.

*p <0.05
