# Supplementary material for: Association of High-Density Lipoprotein Cholesterol with Macular Structure in Nonglaucomatous Individuals
Source: Ophthalmol Sci. 2026 Jan 14;6(3):101073. doi: 10.1016/j.xops.2026.101073 (PMC12907079; doi:10.1016/j.xops.2026.101073)
Supplement: Table S4 [file mmc5.pdf]

Supplementary Table S4. Results of the piecewise linear regression analysis of the association between the GCC thickness and HDL-C level, after excluding participants who received treatment for dyslipidemia

Parameters	Univariable analyses		Multivariable analysis	
Segment of HDL-C, mg/dL	Partial regression coefficient (B) (95% CI)	*P*-value	Partial regression coefficient (B) (95% CI)	*P*-value
Segment 1, <60 mg/dL	−0.18 (−0.32, −0.09)	<0.001*	−0.20 (−0.31, −0.09)	0.001*
Segment 2, 60–67 mg/dL	+0.30 (−0.25, +0.76)	0.316	+0.21 (−0.30, +0.72)	0.413
Segment 3, >67 mg/dL	−0.11 (−0.20, −0.03)	0.008*	−0.12 (−0.20, −0.04)	0.005*

HDL-C = high-density lipoprotein cholesterol; GCC = ganglion cell complex; CI = confidence interval.

In the multivariable analyses, they were adjusted for age, intraocular pressure, and axial length.

*p <0.05
